# Supplementary material for: In Vivo Predictive Dissolution and Biopharmaceutic-Based In Silico Model to Explain Bioequivalence Results of Valsartan, a Biopharmaceutics Classification System Class IV Drug
Source: Pharmaceutics. 2024 Mar 13;16(3):390. doi: 10.3390/pharmaceutics16030390 (PMC10974868; doi:10.3390/pharmaceutics16030390)
Supplement: Supplementary file 1 [file pharmaceutics-16-00390-s001.zip › pharmaceutics-2876320-supplementary.pdf]

Supplementary information
Definition of terms

$X_{ud(segment)}$ = undissolved amount in each GIS chamber or intestinal segment

(s=stomach; d=duodenum; j=jejunum)

$X_{d(segment)}$ = dissolved amount in each GIS chamber or intestinal segment

X_{cen} = amount in central compartment

X_{peri} = amount in peripheral compartment

$V_{segment}$ = volume in each GIS chamber or intestinal segment

$V_{segment,0}$ = initial volume in each GIS chamber or intestinal segment

$k_{sec(segment)}$ = secretion rate constant in each GIS chamber or intestinal segment

$C_{end(s)}$ = final concentration in GIS stomach

F_{sys} = systemic availability;

P_{eff}= effective permeability

k_{12} and k_{21}= central to peripheral and peripheral to central distribution coefficients

R= intestinal radious; 1.5 cm;

DF=degree of flatness; 2;

kt=transit constant from jejunum to distal segments: 0.0056 min^{-1} (Matsui et al., 2017)

Parameters Z_s; Z_d; Z_j i.e dissolution coefficients in each compartment and $kpre_s$ precipitation coefficient in stomach.

Mass Equations for GIS

Undissolved drug in the GIS$_{stomach}$:

$$\frac{dX_{ud(s)}}{dt} = -Z_s\left(X_{d(s)} + X_{ud(s)}\right)^{1/3} X_{ud(s)}{}^{2/3}\left(C_{s(s)} - \frac{X_{d(s)}}{V_s}\right) - \left(-\frac{dV_s}{dt} + k_{sec(s)}\right)\left(\frac{X_{ud(s)}}{V_s}\right) + k_{pre(s)}\left(X_{d(s)} - C_{end(s)}V_s\right)$$

Dissolved drug in the GIS$_{stomach}$:

$$\frac{dX_{d(s)}}{dt} = Z_s(X_{d(s)} + X_{ud(s)})^{1/3} X_{ud(s)}{}^{2/3}\left(C_{s(s)} - \frac{X_{d(s)}}{V_s}\right) - \left(-\frac{dV_s}{dt} + k_{sec(s)}\right)\left(\frac{X_{d(s)}}{V_s}\right) + k_{pre(s)}\left(X_{d(s)} - C_{end(s)}V_s\right)$$

Undissolved drug in the GIS$_{duodenum}$:

$$\frac{dX_{ud(d)}}{dt} = -Z_d(X_{d(d)} + X_{ud(d)})^{1/3} X_{ud(d)}{}^{2/3}\left(C_{s(d)} - \frac{X_{d(d)}}{V_d}\right) + \left(-\frac{dV_s}{dt} + k_{sec(s)}\right)\left(\frac{X_{ud(s)}}{V_s}\right) - \left(-\frac{dV_s}{dt} + k_{sec(s)} + k_{sec\,(d)}\right)\left(\frac{X_{ud(d)}}{V_d}\right)$$

Dissolved drug in the GIS$_{duodenum}$:

$$\frac{dX_{d(d)}}{dt} = Z_d(X_{d(d)} + X_{ud(d)})^{1/3} X_{ud(d)}{}^{2/3} \left(C_{s(d)} - \frac{X_{d(d)}}{V_d}\right) + \left(-\frac{dV_s}{dt} + k_{sec(s)}\right) \left(\frac{X_{d(s)}}{V_s}\right) - \left(-\frac{dV_s}{dt} + k_{sec(s)} + k_{sec\,(d)}\right)\left(\frac{X_{d(d)}}{V_d}\right)$$

Undissolved drug in the GIS$_{jejunum}$:

$$\frac{dX_{ud(j)}}{dt} = -Z_j(X_{d(j)} + X_{ud(j)})^{1/3} X_{ud(j)}{}^{2/3} \left(C_{s(j)} - \frac{X_{d(j)}}{V_j}\right) + \left(-\frac{dV_s}{dt} + k_{sec(s)} + k_{sec\,(d)}\right)\left(\frac{X_{ud(d)}}{V_d}\right)$$

Dissolved drug in the GIS$_{jejunum}$:

$$\frac{dX_{d(j)}}{dt} = Z_j(X_{d(j)} + X_{ud(j)})^{1/3} X_{ud(j)}{}^{2/3} \left(C_{s(j)} - \frac{X_{d(j)}}{V_j}\right) + \left(-\frac{dV_s}{dt} + k_{sec(s)} + k_{sec\,(d)}\right)\left(\frac{X_{d(d)}}{V_d}\right)$$

$V_s = V_{s,0} \times e^{-\left(\frac{\ln(2)}{GE}\right)t}$ [L] (if t > 45 min, t = 45)

$V_d = V_{d,0}$ [L]

$V_j = V_{s,0} \times \left(1 - e^{-\left(\frac{\ln(2)}{GE}\right)t}\right) + (k_{sec\,(s)} + k_{sec\,(d)}) \times t$ [L] (if t > 45 min, t = 45)

$k_{sec\,(s)} = 0.001$ [L/min] (if t > 45 min, $k_{sec\,(s)} = 0$)

$k_{sec\,(d)} = 0.001$ [L/min] (if t > 45 min, $k_{sec\,(d)} = 0$)

Mass Equations for in vivo plasma predictions

Undissolved drug in the stomach:

$$\frac{dX_{ud(s)}}{dt} = -Z_s\left(X_{d(s)} + X_{ud(s)}\right)^{1/3} X_{ud(s)}{}^{2/3}\left(C_{s(s)} - \frac{X_{d(s)}}{V_s}\right) - \left(-\frac{dV_s}{dt} + k_{sec(s)}\right)\left(\frac{X_{ud(s)}}{V_s}\right) + k_{pre(s)}\left(X_{d(s)} - C_{end(s)}V_s\right)$$

Dissolved drug in the stomach:

$$\frac{dX_{d(s)}}{dt} = Z_s\left(X_{d(s)} + X_{ud(s)}\right)^{1/3} X_{ud(s)}{}^{2/3}\left(C_{s(s)} - \frac{X_{d(s)}}{V_s}\right) - \left(-\frac{dV_s}{dt} + k_{sec(s)}\right)\left(\frac{X_{d(s)}}{V_s}\right) - k_{pre(s)}\left(X_{d(s)} - C_{end(s)}V_s\right)$$

Undissolved drug in the duodenum:

$$\frac{dX_{ud(d)}}{dt} = -Z_d\left(X_{d(d)} + X_{ud(d)}\right)^{1/3} X_{ud(d)}{}^{2/3}\left(C_{s(d)} - \frac{X_{d(d)}}{V_d}\right) + \left(-\frac{dV_s}{dt} + k_{sec(s)}\right)\left(\frac{X_{ud(s)}}{V_s}\right) - \left(-\frac{dV_s}{dt} + k_{sec(s)} + k_{sec\,(d)}\right)\left(\frac{X_{ud(d)}}{V_d}\right)$$

Dissolved drug in the duodenum:

$$\frac{dX_{d(d)}}{dt} = Z_d\left(X_{d(d)} + X_{ud(d)}\right)^{1/3} X_{ud(d)}{}^{2/3}\left(C_{s(d)} - \frac{X_{d(d)}}{V_d}\right) + \left(-\frac{dV_s}{dt} + k_{sec(s)}\right)\left(\frac{X_{d(s)}}{V_s}\right) - \left(-\frac{dV_s}{dt} + k_{sec(s)} + k_{sec(d)}\right)\left(\frac{X_{d(d)}}{V_d}\right) - \frac{2DF}{R} X_{d(d)} P_{eff}$$

Undissolved drug in the jejunum:

$$\frac{dX_{ud(j)}}{dt} = -Z_j\left(X_{d(j)} + X_{ud(j)}\right)^{1/3} X_{ud(j)}{}^{2/3}\left(C_{s(j)} - \frac{X_{d(j)}}{V_j}\right) + \left(-\frac{dV_s}{dt} + k_{sec(s)} + k_{sec\,(d)}\right)\left(\frac{X_{ud(d)}}{V_d}\right) - k_t X_{ud(j)}$$

Dissolved drug in the jejunum:

$$\frac{dX_{d(j)}}{dt} = Z_j\left(X_{d(j)} + X_{ud(j)}\right)^{1/3} X_{ud(j)}{}^{2/3}\left(C_{s(i)} - \frac{X_{d(j)}}{V_j}\right) + \left(-\frac{dV_s}{dt} + k_{sec(s)} + k_{sec\,(d)}\right)\left(\frac{X_{d(d)}}{V_d}\right) - \frac{2DF}{R} X_{d(j)} P_{eff} - k_t X_{d(j)}$$

Drug in the central compartment:

$$\frac{dX_{cen}}{dt} = \left(\frac{2DF}{R} X_{d(d)} P_{eff} + \frac{2DF}{R} X_{d(j)} P_{eff}\right) F_{sys} - k_{12} X_{cen} + k_{21} X_{peri} - k_e X_{cen}$$

Drug in the peripheral compartment:

$$\frac{dX_{peri}}{dt} = k_{12}X_{cen} - k_{21}X_{peri}$$

Plasma drug concentration:

$$C_p = \frac{X_{cen}}{V_c}$$

References:

Matsui, K., Tsume, Y., Takeuchi, S., Searls, A., & Amidon, G. L. (2017). Utilization of Gastrointestinal Simulator, an in Vivo Predictive Dissolution Methodology, Coupled with Computational Approach To Forecast Oral Absorption of Dipyridamole. *Molecular Pharmaceutics*, *14*(4), 1181–1189. https://doi.org/10.1021/acs.molpharmaceut.6b01063
